# P026 Catastrophic antiphospholipid syndrome occurring two months after a surgical herald event in a patient with quiescent systemic lupus erythematosus and stable antiphospholipid syndrome on therapeutic warfarin

**DOI:** 10.1093/rap/rkag113.056

**Published:** 2026-11-03

**Authors:** Alexandra Godlee, Charlotte Talbot, Philip Nicolson, Holly Merris, Megan Rutter

**Affiliations:** University Hospitals Birmingham Foundation NHS Trust, Birmingham, United Kingdom; Sandwell and West Birmingham Hospitals NHS Trust, Birmingham, United Kingdom; University Hospitals Birmingham NHS Foundation Trust, Birmingham, United Kingdom; University Hospitals Birmingham NHS Foundation Trust, Birmingham, United Kingdom; Sandwell and West Birmingham Hospitals NHS Trust, Birmingham, United Kingdom

## Abstract

**Introduction:**

Catastrophic antiphospholipid syndrome (CAPS) is a life-threatening variation of antiphospholipid syndrome (APS) where mortality has been estimated to exceed 50%. Thrombotic events can occur at venous, arterial and microvascular level, contributing to multi-organ failure. CAPS can occur as a primary manifestation of APS or can occur in those with known APS and those with co-existing systemic lupus erythematous (SLE). CAPS is most commonly seen in those who are triple positive for APS-associated antibodies and triggers include subtherapeutic anticoagulation, infection, pregnancy, surgery or a flare of SLE. CAPS is rare, affecting approximately 1% of patients with APS.

**Case description:**

Our patient was diagnosed with APS aged 33 following a transient ischaemic attack. Blood tests revealed triple positivity for APS-associated antibodies, and she was noted to have had a history of multiple miscarriages. Immunology tests were positive for dsDNA with low complement, and she gave a history of Raynaud’s, rash and arthralgia in keeping with SLE. Her lupus was managed with oral steroids, azathioprine and hydroxychloroquine. After several years she achieved remission and was weaned from immunosuppressive therapy. Therapeutic warfarin was continued to maintain an INR of 3-4. She remained stable for nearly a decade.

In 2026 our patient was listed for mitral valve replacement surgery secondary to Rheumatic heart disease. During the perioperative period, warfarin was paused and she was maintained on low-molecular-weight heparin.

Her post-operative path was complicated by thrombocytopenia, and liver and adrenal infarcts with superior mesenteric artery thrombus. Warfarin was re-introduced and she was discharged.

Two months later the patient was readmitted with fever and a palpable malar rash. She had felt unwell since her discharge with arthralgia, abdominal pain and Raynaud’s. SLE immunology was unremarkable with normal complement levels and dsDNA. She was initially treated for infection however within 24 hours of admission, she rapidly deteriorated into multiple organ failure requiring intubation with renal and ionotropic support. Echocardiogram showed global ventricular dysfunction. The blood film did not show overt features of thrombotic microangiopathy, however the multidisciplinary team suspected CAPS and plasmapheresis was commenced within hours. Over the subsequent days she was stabilised with plasmapheresis, methylprednisolone and rituximab. Anticoagulation was maintained with unfractionated heparin. She made a remarkable recovery and was discharged from intensive care after 9 days with recovered renal and cardiac function. She is left with a significant psychiatric burden from her admission.

**Discussion:**

This case highlights the diagnostic and therapeutic challenges of CAPS occurring in a patient with longstanding stable APS syndrome and SLE. Catastrophic APS is a rare but life-threatening manifestation of APS characterised by rapidly progressive, widespread thrombotic microangiopathy leading to multiorgan failure.

Infection, surgery, interruption of anticoagulation and disease flares are recognised precipitating factors, several of which were present in this patient. Perioperative cessation of warfarin for mitral valve replacement, despite bridging with low molecular weight heparin, may have contributed to the initial thrombotic complications, while the subsequent inflammatory response to major cardiac surgery likely acted as an additional trigger.

A notable feature of this case was the difficulty in distinguishing CAPS from an SLE flare or infection. Despite the presence of fever, malar rash, arthralgia and Raynaud’s phenomenon, lupus serology remained quiescent with normal complement levels and anti-dsDNA titres, making active SLE less likely. Similarly, the recent cardiac surgery appropriately prompted empirical treatment for endocarditis. However, the patient’s rapid deterioration with recurrent visceral infarction, severe coagulopathy and biventricular failure was more consistent with CAPS, emphasising the importance of maintaining a high index of suspicion in high-risk patients.

Early recognition and prompt initiation of treatment were central to this patient’s favourable outcome. Plasmapheresis was commenced within hours of intensive care admission and combined with high-dose corticosteroids, therapeutic anticoagulation using unfractionated heparin guided by anti-Xa monitoring, and rituximab. This multimodal approach aligns with current recommendations for severe or refractory CAPS and likely limited irreversible organ damage. Her recovery of cardiac and renal function demonstrates the potential reversibility of even profound organ dysfunction when treatment is initiated promptly. Finally, this case highlights the substantial psychological morbidity experienced by survivors of critical illness and reinforces the importance of incorporating long-term multidisciplinary follow-up, including specialist psychological support, into recovery after CAPS.

**Key learning points:**

1. CAPS can occur in patients with quiescent SLE and stable APS

2. The inflammatory burden of major cardiac surgery with interruption of warfarin dosing was a likely herald event occurring two months prior to deterioration

3. Prompt recognition and treatment of CAPS undoubtedly contributed to the favourable outcome

4. This case highlights the psychological morbidity experienced by survivors of critical illness and reinforces the importance of multidisciplinary follow up including psychological support.

